# Select cognitive deficits in Vasoactive Intestinal Peptide deficient mice

**DOI:** 10.1186/1471-2202-9-63

**Published:** 2008-07-10

**Authors:** Dipesh Chaudhury, Dawn H Loh, Joanna M Dragich, Arkady Hagopian, Christopher S Colwell

**Affiliations:** 1 Department of Psychiatry and Biobehavioral Sciences, University of California – Los Angeles, 760 Westwood Plaza, Los Angeles, California 90024-1759, USA; 2 Department of Neurobiology and Behavior, W257 Seeley G Mudd Bioscience Wing, Cornell University, Ithaca, New York, 14850, USA

## Abstract

**Background:**

The neuropeptide vasoactive intestinal peptide (VIP) is widely distributed in the adult central nervous system where this peptide functions to regulate synaptic transmission and neural excitability. The expression of VIP and its receptors in brain regions implicated in learning and memory functions, including the hippocampus, cortex, and amygdala, raise the possibility that this peptide may function to modulate learned behaviors. Among other actions, the loss of VIP has a profound effect on circadian timing and may specifically influence the temporal regulation of learning and memory functions.

**Results:**

In the present study, we utilized transgenic VIP-deficient mice and the contextual fear conditioning paradigm to explore the impact of the loss of this peptide on a learned behavior. We found that VIP-deficient mice exhibited normal shock-evoked freezing behavior and increases in corticosterone. Similarly, these mutant mice exhibited no deficits in the acquisition or recall of the fear-conditioned behavior when tested 24-hours after training. The VIP-deficient mice exhibited a significant reduction in recall when tested 48-hours or longer after training. Surprisingly, we found that the VIP-deficient mice continued to express circadian rhythms in the recall of the training even in those individual mice whose wheel running wheel activity was arrhythmic. One mechanistic explanation is suggested by the finding that daily rhythms in the expression of the clock gene *Period2 *continue in the hippocampus of VIP-deficient mice.

**Conclusion:**

Together these data suggest that the neuropeptide VIP regulates the recall of at least one learned behavior but does not impact the circadian regulation of this behavior.

## Background

The neuropeptide vasoactive intestinal peptide (VIP) is expressed in specific subpopulations of neurons in the central and peripheral nervous system. Two receptors, encoded by distinct genes, bind VIP with high affinity: VPAC_1_R and VPAC_2_R [1-3]. VIP and its receptors are expressed in those brains regions thought to be involved in learned behaviors, including the hippocampus, cortex, amygdala and hypothalamus e.g. [4-6]. While the physiological actions of this neuropeptide have not been extensively studied, VIP regulates synaptic transmission [e.g. [7,8]] and intrinsic membrane currents [e.g. [9-12]]. Thus, this neuropeptide can be a potent modulator of neural activity and function in specific circuits in the adult nervous system.

Previous pharmacological studies have raised the possibility that VIP modulates learning and memory processes. The central administration of VIP causes marked impairment in passive avoidance and spatial memory [13-17]. Inhibition of VIP by use of antagonists also affects spatial learning [18]. Removal of VIP-producing cells by expression of a chimeric VIP-diptheria toxin gene caused learning deficits [19]. Furthermore, VIP agonists protect against the impaired spatial learning observed in experimental Alzheimer mouse models [20,21]. Mechanistically, VIP regulates the secretion and expression of neurotrophic factors [22-26] and VPAC receptors are potent activators of the adenylyl cyclase/protein kinase A cascade [27] that has been implicated in the regulation of long-term memory formation [e.g. [28,29]]. Interestingly, recent work found that treatment of pregnant mice with a VIP antagonist caused cognitive deficits in the male offspring and lead to the suggestion that these mice may serve as a model of autism [30]. Together, this literature is consistent with hypothesis that VIP signaling could regulate learned behavior in the adult.

There is strong evidence that VIP and the VPAC_2_R are critical for the normal functioning of the circadian system. Early studies on young rats showed breakdown in locomotor rhythms following application of VIP antagonists [31]. More recent studies come via the development of transgenic mice lacking VIP [32] or VPAC_2_R [33]. All of the VIP- and VPAC_2_R-deficient mice exhibit disruptions in their ability to express a coherent circadian rhythm in constant conditions although the extent of the arrhythmic phenotype varies from animal to animal. At a cellular level, VIP- and VPAC_2_R-deficient mice fail to exhibit the midday peak in electrical activity that is characteristic of impulse rhythms from suprachiasmatic nuclei (SCN) brain slices [34-36]. Together, these data indicate that VIP and VPAC_2_R are critical for the generation of behavioral rhythmicity in mice and that the deficit occurs at the level of the SCN. This data also serves to point out the utility of transgenic models in the exploration of the role of VIP in behavior.

In the present study, we used VIP-deficient mice to determine the role of this peptide in the regulation of contextual fear conditioning. We first determined whether VIP-deficient mice exhibited deficits in shock-evoked freezing behavior or corticosterone response. Next, we determined whether these mice exhibited deficits in acquisition or recall of contextual fear response. Furthermore, we examined whether the loss of VIP influenced the daily and circadian rhythms in the recall of the conditioned fear response. Finally, we determined whether daily rhythms in the expression of the clock gene *Period 2 *could be measured in the hippocampus of VIP-deficient mice.

## Results

### Acute freezing behavior in WT and VIP-deficient mice

**Figure 1:**
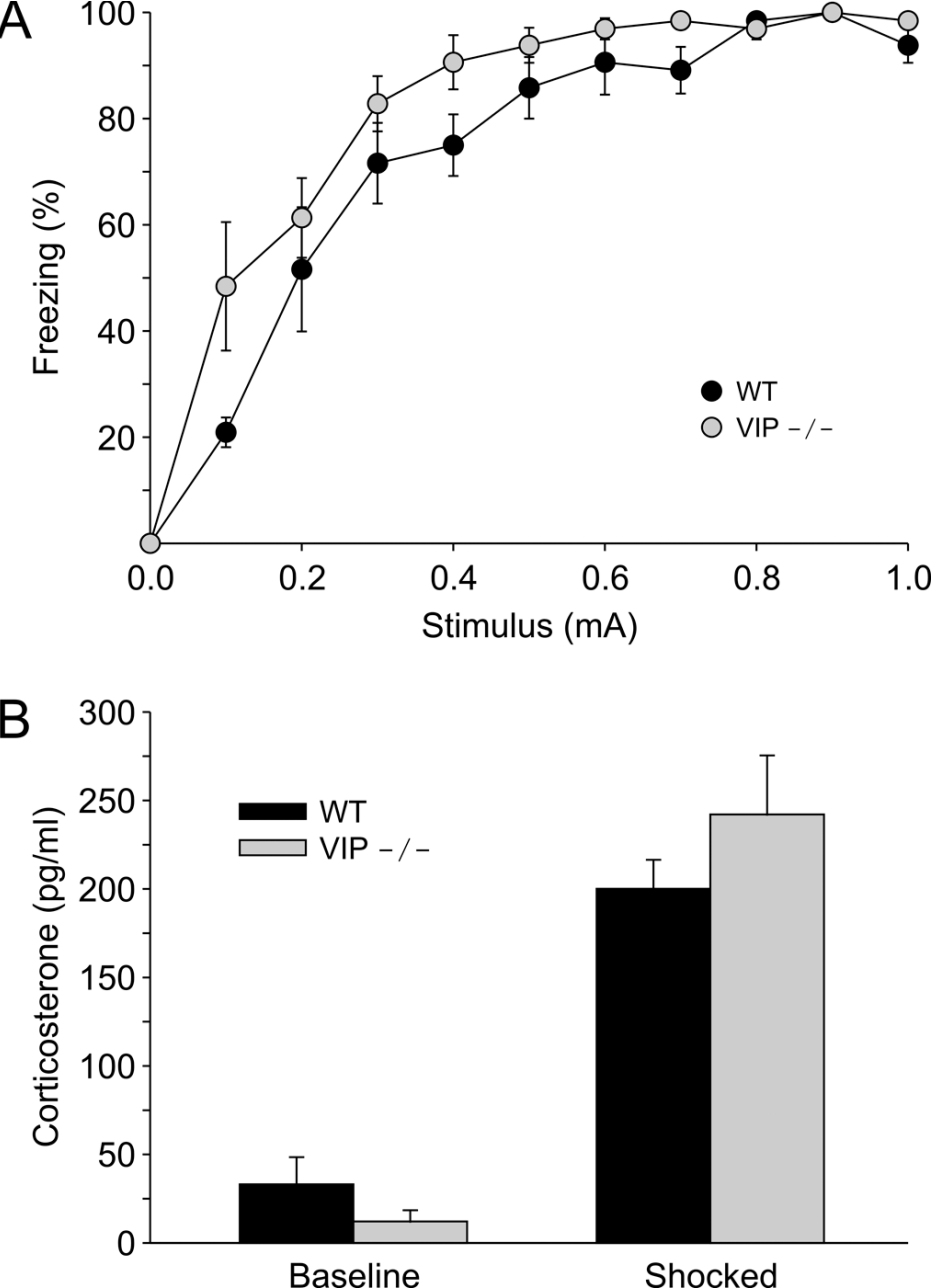
**The loss of VIP does not impact shock-evoked freezing or corticosterone response**. **A) **WT and VIP-deficient mice were directly shocked with progressively increasing stimulus intensities and the freezing response measured. The measurement of these acute fear responses were conducted in the early day (ZT 3). The VIP-deficient mice exhibited more freezing in response to the lowest intensity shock (0.1 mA) but there were no differences between the genotypes at the other shock intensities. **B**) Corticosterone response to acute stress evoked by foot-shock (0.2 mA applied twice) in the early day (ZT3). Both WT and VIP-deficient mice displayed a significant induction of corticosterone in response to acute stress (*P *< 0.05). There were no significant differences between the two genotypes in the baseline or the evoked corticosterone levels.

We first determined if VIP-deficient mice exhibited deficits in sensitivity to foot-shock by testing the direct shock-evoked fear response at ZT3 (day). WT and VIP-deficient mice were exposed to shocks of a range of intensities (0.1 to 1 mA, duration) and the freezing response measured (Fig. 1A). Overall, the freezing response to the shock were higher in the VIP-deficient mice (RM-ANOVA: *P *< 0.05) compared to WT controls. The VIP-deficient mice exhibited significantly (t-test: t = 3, *P *< 0.01) more freezing at the lowest intensity (0.1 mA). For the remainder of the study, we used the lowest intensity of shock (0.2 mA) that did not produce any significant differences between the genotypes. Thus, the VIP-deficient mice do not exhibit defects in shock-evoked freezing behavior.

### Acute corticosterone response in WT and VIP-deficient mice

The concentration of circulating corticosterone is actively regulated as part of the stress response exhibited by mice. We sought to determine whether the endocrine response to foot-shock (0.2 mA) was altered in VIP-deficient mice. For these experiments, we examined corticosterone response to a mild foot-shock in WT and VIP-deficient mice in early day (ZT 3, *n *= 3 per group). WT mice subjected to foot-shock exhibited significantly higher concentrations (t-test: t = -4.0, *P *< 0.05) of serum corticosterone than unstressed mice (Fig. 1B), and VIP-deficient mice showed a similar increase in corticosterone concentration upon exposure to acute stress (t-test: t = -3.3, *P *< 0.05). There were no significant differences in the baseline or stress-evoked serum corticosterone concentrations between WT and VIP-deficient mice, suggesting the induction of corticosterone in response to stress is intact in VIP-deficient mice.

### Acquisition in VIP KO mice following contextual fear training

**Figure 2:**
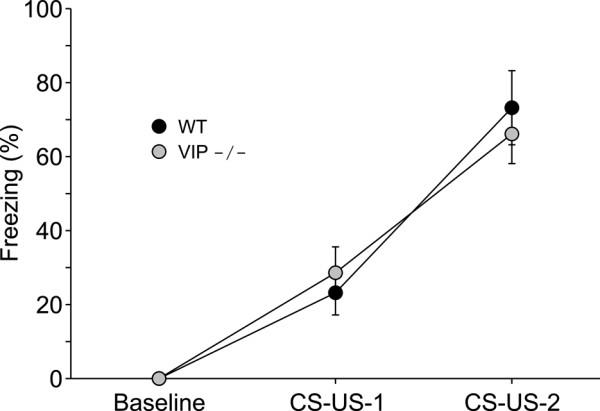
**The loss of VIP does not impact acquisition of contextual fear conditioning**. WT and VIP-deficient mice were trained in the day (ZT 3). Conditioning consisted of two context (CS) and foot shock (US) pairings. Percent freezing following each context-shock pairing was compared between WT (black circles) and VIP-deficient (grey circles) mice (*n *= 8 per group). There was no difference between the two genotypes in the acquisition of the conditioned fear response.

Given that VIP is expressed in brain regions implicated in learning, including the hippocampus, we sought to determine if learning was altered in VIP-deficient mice. For these experiments, we examined the acquisition of contextual fear conditioning in WT and VIP-deficient mice at ZT 3 using 2 CS-US pairing with 0.2 mA shock (*n *= 8 per group). Both WT and VIP-deficient mice learned the task (Fig. 2) and there were no significant differences in the acquisition between the two genotypes (t-test: CS-US-1: t = 0.5, *P *= 0.63; CS-US-2: t = -0.4, *P *= 0.73). With a second set of animals, we examined the performance of the two genotypes with a stronger training protocol that consisted of 3 CS-US pairings with 0.2 mA shock (*n *= 10 per group). Again, statistical analysis did not show any significant difference in the degree of acquisition between VIP-deficient and WT mice (t-test: CS-US-1: t = -0.8, *P *= 0.43; CS-US-2: t = -0.4, *P *= 0.68; CS-US-3: t = 1.1, *P *= 0.32). Thus, the loss of VIP does not influence the ability of mice to acquire contextual fear conditioning.

### Contextual recall 24, 48, 72-hrs following training

**Figure 3:**
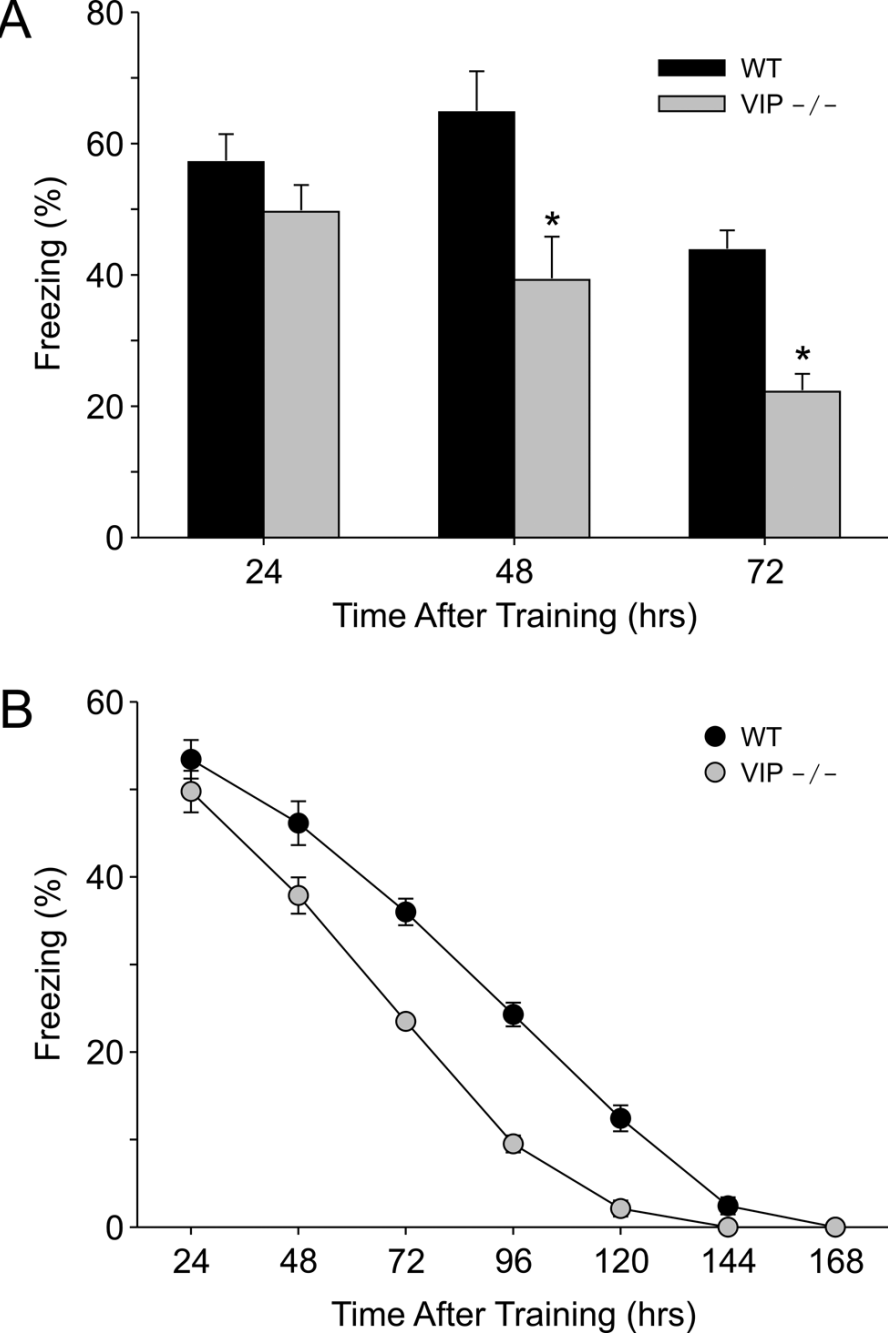
**The loss of VIP impaired recall of contextual fear conditioning**. **A) **Groups of VIP-deficient and WT mice (*n *= 7–8 per group) were trained on the contextual fear conditioning (2 CS-US pairings) at ZT 3. Mice were then tested for the recall of fear conditions one, two, or three days after training with each animal only being tested once. There was no difference the acquisition of the conditioned fear response during training and all of the groups (24-, 48-, 72-hr time points) exhibited a robust recall of context as measured by freezing behavior. VIP-deficient mice (grey bars) exhibited significant reductions in recall compared to WT mice (black bars) when measured at 48- (*P *< 0.05) and 72-hrs (*P *< 0.001) post-training. At one day after training, there was no significant difference in the percent freezing between the genotypes. **B) **In another experiment, VIP-deficient (grey circles) and WT (black circles) mice were trained and then tested for recall once per day for 7 days (*n *= 8 per group). Training and testing were conducted at ZT 3. The VIP-deficient mice exhibited significant reductions in recall compared to WT controls (*P *< 0.01). Post-hoc analysis (t-tests) indicated significant differences between the genotypes when measured at 48- (*P *= 0.02), 72- (*P *< 0.001), 96- (*P *< 0.001) and 120-hrs (*P *< 0.001) post-training.

Next, we examined if the recall of the contextual fear conditioning is altered in VIP-deficient mice. Groups of VIP-deficient and WT mice (*n *= 7–8 per group) were trained on the weak version of contextual fear conditioning (2CS-US pairings) at ZT 3. Mice were then tested for the recall of fear conditions at either 24-, 48-, or 72-hrs after training (Fig. 3A) with each animal being tested only once. There was no difference in  the acquisition of the conditioned fear response, with the group means  all between 64 and 70% freezing. All of the groups (24-, 48-, 72-hr time points) exhibited a robust recall of context as measured by freezing behavior (Fig. 3A). Overall, the VIP-deficient mice exhibited significant reductions in recall compared to WT controls (ANOVA: F = 9.2, *P *< 0.001). Post-hoc analysis (t-tests) indicated that VIP-deficient mice exhibited significant reductions in recall compared to WT mice when measured at 48- (t = 2.6, *P *< 0.05) and 72-hrs (t = 5.4, *P *< 0.001) post-training. At 24-hrs post training, there was no significant difference in contextual recall between the genotypes. To further explore this deficit in longer-term recall, additional groups of WT and mutant mice (*n *= 8 per group) were trained on contextual fear conditioning at ZT 3 and then tested once per day for 7 days (Fig. 3B). Overall, the VIP-deficient mice exhibited significant reductions in recall compared to WT controls (RM ANOVA: F = 12.6, *P *< 0.01). Post-hoc analysis (t-tests) indicated significant differences between the genotypes when measured at 48- (t = 2.6, *P *= 0.020), 72- (t = 8.0, *P *< 0.001), 96- (t = 9.3, *P *< 0.001) and 120-hrs (t = 6.2, *P *< 0.001) post-training. At 24-hrs post training, there was no significant difference in contextual recall between the WT and VIP-deficient mice. By 144 hrs, the conditioned fear response had extinguished in both groups. Together, this data indicates that the VIP-deficient mice exhibited deficits in the recall of contextual fear conditioning when tested at least 48-hrs after training.

### Rhythm in contextual recall under LD conditions

**Figure 4:**
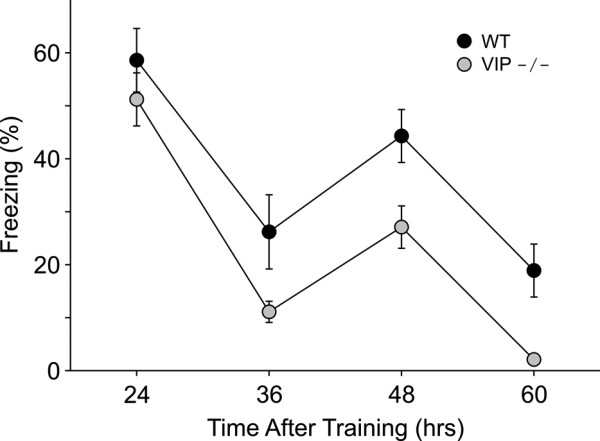
**VIP-deficient mice exhibit a daily rhythm in the recall of contextual fear conditioning**. Groups of WT (black circles) and VIP-deficient (grey circles) mice were trained at ZT 3 and then tested 24-, 36-, 48- and 60-hrs after training (*n *= 8 per group). There was no difference in the acquisition between the genotypes (WT: 66 ± 5% freezing; VIP KO: 69 ± 7% freezing). Both genotypes showed evidence for diurnal rhythmicity in recall with significant (*P *< 0.05) peaks observed at 24- and 48-hrs after training. However, compared to WT mice, the VIP-deficient mice exhibited significantly (*P *< 0.05) lower recall when measured 36-, 48-, or 60-hrs after training.

We previously demonstrated that the recall of contextual recall expresses robust circadian variation [37] and that the circadian system of VIP-deficient mice is compromised [32]. Therefore, we first sought to determine if VIP-deficient mice exhibit a diurnal rhythm in the recall of contextual fear conditioning. Groups of WT and VIP-deficient mice (*n *= 8 per group) were trained at ZT3. The mice were then tested at several intervals after training: 24-, 36-, 48-, 60-hrs (Fig. 4). While there was no difference in the acquisition between the genotypes (WT: 66 ± 5% freezing; VIP KO: 69 ± 7% freezing), the VIP-deficient mice exhibited significant reductions in recall compared to WT mice (RM ANOVA: F = 12.6, *P *< 0.01). Compared to WT, VIP-deficient mice exhibited significantly less freezing at 36-hr (t = 2.1, *P *< 0.05), 48-hr (t = 2.6, *P *< 0.01), and 60-hr (t = 2.5, *P *< 0.01) after training. For both genotypes, the recall varied with time (RM-ANOVA: *P *< 0.001) with post-hoc analysis (Tukey test) indicating that freezing at 24- and 48-hrs after training was significantly (*P *< 0.05) higher than the freezing at 36- and 60-hrs for both strains of mice. Thus, in LD conditions, both WT and VIP-deficient mice exhibited a diurnal rhythm in recall.

### Rhythm in contextual recall in behaviorally rhythmic and arrhythmic VIP KO mice

**Figure 5:**
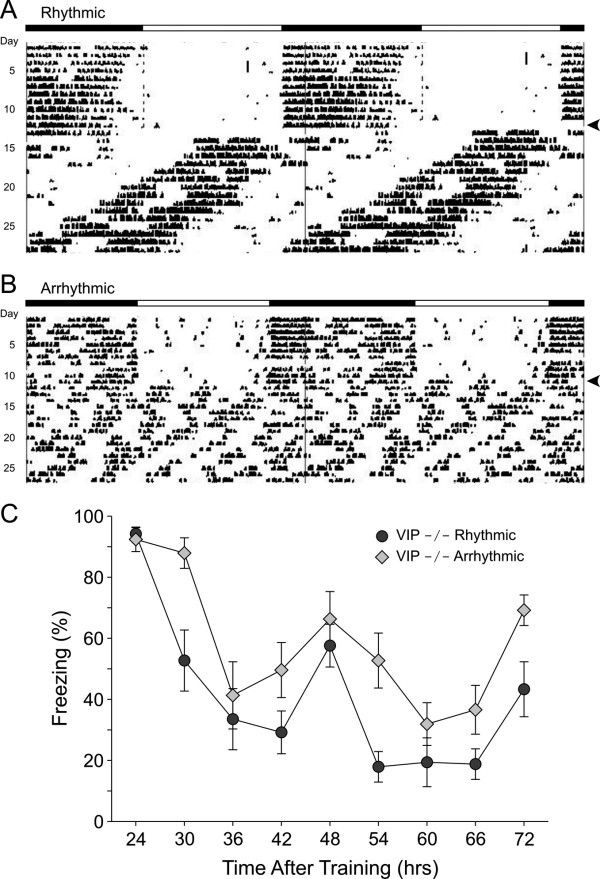
**VIP-deficient mice that have arrhythmic patterns of wheel-running activity exhibit a circadian rhythm in the recall of contextual fear conditioning**. **A) **Example of wheel-running activity from a VIP-deficient mouse that maintained a circadian rhythm in wheel-running activity. Bars at top of panel indicate the LD cycle. The locomotor activity is double-plotted as an aid to visual analysis. The mouse was placed into DD on the day indicted by the arrow on the right. **B) **Example of wheel-running activity from a VIP-deficient mouse that was judged to by arrhythmic on a circadian time-scale by visual analysis and periodogram. **C) **Both rhythmic and arrhythmic VIP-deficient mice were trained at CT 3 and then tested every 6-hrs between 24- and 72-hrs after training. These experiments were conducted in constant darkness to measure the endogenous rhythmicity. For the rhythmic mice (dark grey circles), the onset of locomotor activity was used to estimate phase while, for the arrhythmic mice (grey diamonds), the time of the prior LD cycle was used. Both groups of VIP-deficient mice exhibited circadian rhythms in the recall of fear conditioning with significant (*P *< 0.05) peaks 24-, 48-, and 72-hrs post training.

The next set of experiments was designed to determine whether VIP-deficient mice exhibit circadian rhythms in recall. For these experiments, WT and VIP-deficient mice were held in constant conditions in cages with running wheels. WT mice all exhibited clear circadian rhythms in wheel-running activity. While all of the VIP-deficient mice exhibited disrupted rhythms [32], some of the mutant mice are arrhythmic while others continue to express circadian rhythms in locomotor activity (Fig. 5A,B). For these experiments, groups of rhythmic and arrhythmic VIP-deficient mice (*n *= 8 per group) were trained at CT3. For the rhythmic VIP-deficient mice, CT3 (subjective day) was estimated based on wheel running activity. For the arrhythmic VIP-deficient mice, CT 3 was estimated based on the activity projected from the prior LD cycle. Due to the lack of regular activity onset in these mice, a 6 hr phase advance was assumed and tau was also assumed to be around 22 hr based on the activity patterns of the rhythmic VIP-/- mice. Both groups of mice were trained in constant darkness at CT3 using a stronger training protocol consisting of 3 CS-US pairings. There was no difference in the acquisition of conditioning between the 2 groups (Rhythmic: 94 ± 4%; Arrhythmic: 91 ± 5%). The mice were then tested for recall 24-hrs after training and every 6-hrs subsequently for a total of 2 days (Fig. 5C). For both rhythmic and arrhythmic groups, the recall varied with time (RM ANOVA: *P *< 0.001) with post-hoc analysis (Tukey test) indicating that freezing at 24-, 48- and 72-hrs after training was significantly (*P *< 0.05) higher than the freezing at the other intervals. Thus, VIP-deficient mice that exhibited arrhythmic wheel-running activity still express circadian rhythms in the recall of contextual fear conditioning.

### Period2 message is rhythmically expressed in the hippocampus of VIP-deficient mice

**Figure 6:**
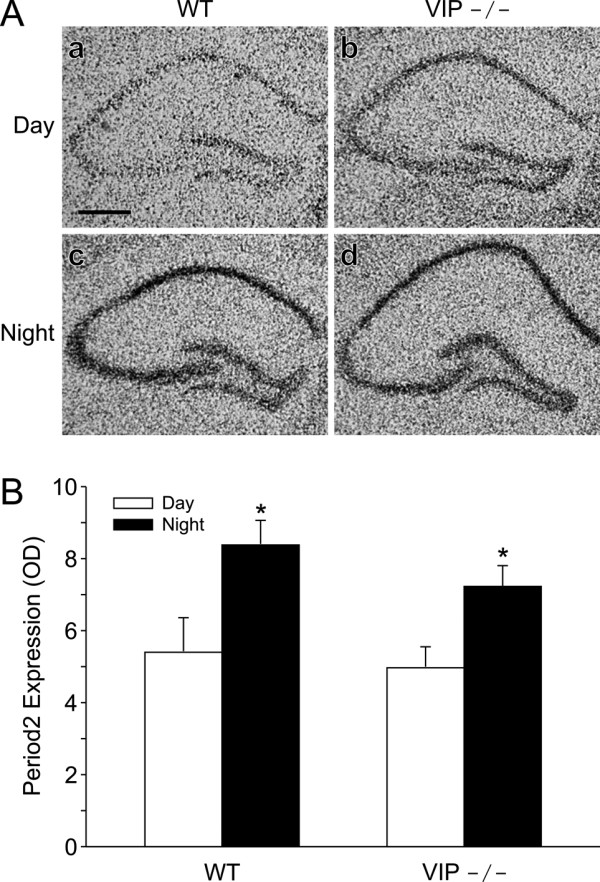
**A circadian rhythm in *Period2 *mRNA in the hippocampus of VIP-deficient mice as measured by ISH**. **A) **Film images of ISH for *Period2 *on tissue sections taken from WT and VIP-deficient mice sacrificed during the subjective day (CT10; a, b) and subjective night (CT 23; c, d). The scale bar equals 0.5 mm. **B) **Optical density measurements of *Period2 *labeling in the hippocampus of WT and VIP-deficient mice during the subjective day (CT 10, white bars) and subjective night (CT 23, black bars). The *Period2 *hybridization signal was significantly (*P *< 0.05) higher in the subjective night in the hippocampus of both lines of mice.

The genes responsible for the generation of circadian oscillations are expressed in brain regions outside of the suprachiasmatic nucleus, including the hippocampus [38,39]. This rhythm in gene expression in the hippocampus may be critical for the rhythms in recall that we and others have observed under various contextual memory tasks. In order to determine if the message coding for *Period2 *is rhythmic in the hippocampus of VIP-deficient mice, we used *in situ *hybridization (ISH) to measure *Period2 *message in the hippocampus of mice at subjective day and night (CT 10 and 23; Fig. 6A). Expression of *Period2 *mRNA was observed throughout the rostrocaudal extent of the hippocampus and was largely restricted to the pyramidal cell layers and the Dentate Gyrus. In the hippocampus of the VIP-deficient mice, the mean optical density (OD) of *Period2 *labeling was significantly higher at subjective day than in the subjective night (Fig. 6B; t-test: t = -2.5, *P *= 0.03). The *Period2 *expression did not significantly vary between VIP-deficient and WT mice. Among the hippocampal subregions, the CA3 pyramidal cell layers showed the most robust circadian variation in labeling (CA3 CT 10: 1.3 ± 0.2 OD, *n *= 9; CA3 CT 23: 2.2 ± 0.2 OD, *n *= 6; t-test: t = -2.92, *P *< 0.01). The *Period2 *sense probe did not exhibit labeling in the brain under identical hybridization conditions (data not shown). These data demonstrate at least one clock gene is rhythmically expressed in the hippocampus of VIP-deficient mice and raise the possibility that extra-SCN circadian oscillators may drive the rhythms in recall in these mice.

## Discussion

The neuropeptide VIP and its receptors are expressed in regions of the brain implicated in the control of learned behaviors including the hippocampus, cortex, amygdala and hypothalamus [e.g. [4-6]]. In these regions, VIP is most commonly expressed in GABAergic interneurons [e.g. [40-43]] that may use this peptide to communicate with specific post-synaptic targets. However, it is equally plausible that VIP functions more as a paracrine signal acting at sites more distant than just the adjacent postsynaptic neurons. While the physiological actions of this neuropeptide have not been extensively studied, VIP regulates synaptic transmission [e.g. [7,8]] and intrinsic membrane currents [e.g. [9-12]]. Thus, this neuropeptide can be a potent modulator of neural activity and function in specific circuits in the adult nervous system. Given this anatomical distribution and potential physiological functions in both the developing and mature nervous system, the loss of VIP may well have been expected to have global influences on learning and memory. However, the present study found that the deficits in the VIP-deficient mice were quite selective. Deficits were not observed in foot shock-evoked fear behavior. This demonstrates that the basic sensory and motor processes controlling this behavior are intact in the VIP-deficient mice. Similarly, the shock-evoked corticosterone response was intact in these mutant mice. Given that VIP is expressed in the adrenals and the circadian system that regulate corticosterone secretion, we felt that it was important to confirm that this aspect of the stress response was functioning in the VIP-deficient mice. Finally, we observed no deficits in the VIP-deficient mice in the acquisition of fear conditioning or in the recall measured at 24-hrs after training. Clearly, the circuitry involved in the acquisition of contextual fear conditioning is either not regulated by VIP or there is compensation for the loss of this neuropeptide in these transgenic mice.

The recall of the contextual fear conditioning was affected by the loss of VIP. These recall deficits were seen in three independent experiments including those mice only tested for recall a single time (Fig. 3A), those tested daily (Fig. 3B), and those tested every 12-hrs (Fig. 4). These results fit with previous pharmacological studies that have raised the possibility that VIP plays a physiological role in the modulation of learning and memory. For example, administration of VIP into rat hippocampus following training on a passive avoidance task induced recall in rats tested 24-hrs later, while administration of a VIP antagonist enhanced retention [14]. Other studies have also found evidence that the central administration of VIP impairs recall in passive avoidance learning [13,17]. Spatial learning as measured by the water maze was also impaired by the ventricular administration of this neuropeptide [15,16]. In contrast, one study reported that the intracerebral administration of a VIP receptor antagonist, but not VIP itself, inhibited performance on the Morris water maze [18]. In addition, learning deficits in mice carrying a chimeric VIP-diptheria toxin gene have been reported [19]. These transgenic mice lost about 20% of VIP content as measured by radioimmunoassay and exhibited learning deficits as measured by the Morris water maze. Taken together, these studies suggest that abnormally high or low levels of VIP can interfere with the acquisition and recall of specific learned behaviors.

The VIP-deficient mice used in the present study are a traditional transgenic model in which the gene coding for VIP has been inactive throughout development. VIP is expressed early in the fetal brain [44] with VIP binding sites abundant on the floor plate of the neural tube [45]. While not extensively studied in central neurons, VIP is a neurotrophic factor that can regulate neural growth, migration, and process formation [reviewed by [46-48]] and, through these developmental mechanisms, influence the neural circuits involved in learning and memory functions. A recent study examined the consequences of pharmacologically blocking VPAC receptors during embryogenesis and examining potential cognitive deficits in the adult offspring [30]. Male, but not female, treated mice exhibited deficits in contextual fear conditioning and social behavior. The selective set of behavioral deficits coupled with the gender difference led these authors to propose these mice as a model for the behavioral deficits of autism. Like the VIP-deficient mice, these mice treated developmentally with the VPAC antagonist did not show deficits in acquisition or in recall measured 24-hrs after training. The male offspring did show recall deficits when measured 48-hrs after training. Thus, the memory deficits observed in the transgenic animals in the present study could well be due to a loss of VIP early in development.

As described before, there is strong evidence that VIP and the VPAC_2_R are critical for the normal functioning of the circadian system [reviewed by [49]]. Together, these data indicate that VIP and VPAC_2_R are critical for the generation of behavioral rhythms in mice and that the deficit occurs at the level of the SCN. Furthermore, the VPAC_2_R-deficient mice even lose the daily rhythms in clock gene expression that are thought to lie at the heart of the machinery for the generation of daily rhythms [33,50]. Given the overwhelming data that the loss of VIP influences the generation of circadian oscillations, the finding that these mutants showed no apparent deficiency in the diurnal and circadian regulation in recall was unexpected. This disassociation was particularly striking in the case of VIP-deficient mice that exhibited arrhythmic locomotor activity. The 6 hr interval in testing may have occluded small changes in peak recall time, which most likely tracks with the intrinsic free-running period of the animals. However, the frequency of testing was sufficient to show that there remains an optimal time of day, which tracks with the same time of day as the initial training exercise, even in mice with arrhythmic wheel-running activity. This is consistent with the phenomenon of "time-stamping" of learned behavior as observed in hamsters [51,52] and by us in mice [37]. What's more, this time-stamp phenomenon may be independent of the SCN. Lesioning the SCN of rats does not affect the time-stamped training of a T-maze reward task [53]. Previous work has found that these behaviorally arrhythmic VIP-deficient mice also exhibit arrhythmic electrical activity rhythms when measured at the level of the SCN [35]. We now show that these arrhythmic mice exhibited clear rhythms in recall and these rhythms were extremely similar to those exhibited by the behaviorally rhythmic mice. Thus, the loss of circadian function in these arrhythmic VIP-deficient mice had no obvious impact on the rhythms in recall suggesting that a rhythmic SCN may not be necessary for the circadian rhythm in recall. In recent years, it has become clear that many of the "clock genes" are expressed outside of the SCN [e.g. [54,55]]. This raises the possibility that oscillators outside of the SCN may drive the rhythms in recall of learned behaviors. While we did not directly address this possibility in the present study, we did examine the expression of one clock gene, *Period2*, in the hippocampus. We found that levels of this gene continue to show circadian differences in the hippocampus of VIP-deficient mice (Fig. 6). This type of rhythmic expression is at least consistent with the possibility that extra-SCN rhythms in gene expression may be directly tied to the rhythms in recall observed in fear-conditioned mice.

In mammals, neurons in the hippocampus [[38,39], present data], olfactory bulb [56], SCN [e.g. [55]], and other brain regions [54] have now been shown to generate oscillations in circadian gene expression. This pool of data contributes to the view that the circadian system is comprised of multiple oscillatory components, with the role of the SCN being a master timer synchronizing these disparate cell populations [e.g. [57]]. With this new view of clock genes and circadian organization, it becomes critical to determine the tissue-specific function of these genes. Logically, local rhythms in clock gene expression could serve to control the temporal program of gene expression and physiology specific to the hippocampus, as recently demonstrated in liver [58]. However, unfortunately, we still do not know much about the functional significance of clock gene expression outside of the SCN. The VIP-deficient mice with weakened rhythms in SCN electrical activity may represent an advantageous model to explore coupling between different circadian oscillators. Our data with contextual fear conditioning raise the possibility that these rhythms in recall or memory are only weakly coupled to core time-keeping mechanisms in the SCN.

## Conclusion

We found that VIP-deficient mice exhibited normal shock-evoked freezing behavior and increases in corticosterone. Similarly, these mutant mice exhibited no deficits in the acquisition or recall of the fear-conditioned behavior when tested 24-hours after training. The VIP-deficient mice exhibited a significant reduction in recall when tested 48-hours or longer after training. Surprisingly, we found that the VIP-deficient mice continued to express circadian rhythms in the recall of the training even in those individual mice whose wheel running wheel activity was arrhythmic. One mechanistic explanation is suggested by the finding that daily rhythms in the expression of the clock gene *Period2 *continue in the hippocampus of VIP-deficient mice. Together these data suggest that the neuropeptide VIP regulates the recall of at least one learned behavior but does not impact the circadian regulation of this behavior.

## Methods

### Experimental animals

We obtained two-month old male mice lacking the gene encoding for the neuropeptides VIP and PHI that had been backcrossed to the C57BL/6J strain for 12 generations [32]. Wild type (WT) littermates were used when available, but age-matched controls were obtained when necessary. All mice were housed in cages within light-tight chambers with controlled lighting conditions. The experimental protocols used in this study were approved by the UCLA Animal Research Committee and all recommendations for animal use and welfare, as dictated by the UCLA Division of Laboratory Animals and the guidelines from the National Institutes of Health, were followed.

### Training and testing procedure

Contextual fear conditioning was performed using previously published protocols [37]. Mice were entrained to a 12:12 light-dark cycle (LD) for at least one week prior to the start of all experiments (light intensity 36 μW/cm^2 ^≅ 120 lux). Zeitgeber Time 0 (ZT0) corresponds to the start of the light period with experiments typically done at ZT3 (early day). Mice were individually handled for approximately 1 min a day, a week prior to the start of the experiment to reduce the arousal associated with handling. Each day, animals were handled at different times of the day or night to ensure that they did not entrain to handling by the experimenter at a specific time. Animals were handled by taking individuals out of their home cages and placing them on the experimenter's arm. Following entrainment, the animals were trained in separate contextual conditioning cages (28 × 21 × 22 cm: Lafayette Instruments). The chambers were constructed from aluminum (sidewalls) and Plexiglas (rear wall, ceiling, and hinged front door). A total of 4 identical conditioning cages were used that allowed 4 mice to be trained and tested per session. The floor of each cage consisted of 33 stainless steel rods (4 mm diameter, 4 mm apart) connected to a shock scrambler and generator (Master Shock, Lafayette Instruments). To remove any variability in olfactory learning, the inside of each cage was wiped with 0.01% benzaldehyde before the start of each experiment. On the day of training, mice were placed individually into cages and allowed to acclimatize to the new environment (conditioned stimulus, CS) for 3 min after which time animals received a 2 sec footshock (unconditioned stimulus, US).

Depending on whether the animals were trained on a strong or weak training task, the number of CS-US pairing and US intensity was varied. The training protocol normally consisted of 2 CS-US pairings with a 0.2 mA US. In some cases, a stronger training protocol consisting of 3 CS-US pairings with a 0.2 mA US was used. The inter-trial interval was 64 seconds in all protocols. At the end of the last context-shock pairing the mice were left in the cage for a further 64 sec, after which they were returned to the home cages. Animals were placed individually into the same conditioning chamber and left there for 8 min. The behavior of the mice, whether it was freezing or mobile was noted. The training procedures were automatically controlled by a computer using the ABET behavioral software (ABET systems, Lafayette Instruments). Control experiments were also carried out where animals were placed in context cages at times points corresponding to those in experiments of animals tested every 6 or 12 hrs. This 'baseline' freezing level was subtracted from freezing in animals following training and subsequent repeated testing.

Freezing was defined as the complete absence of somatic and motility movements with the exception of respiratory movements. For acquisition and context recall, an 8 sec time sampling procedure was used in which each animal was observed 8 times per min interval (in this case a minute refers to a 64 sec block) and these were averaged to yield an estimate of percentage time freezing. During training, freezing was measured 64 sec before the first CS-US pairing (baseline) and during the 64 sec inter-trial interval immediately after each CS-US pairing, giving 8 observations per mice for baseline and for each subsequent CS-US pairing respectively. For context testing, each animal was observed a total of 64 times. To determine the degree of learning during training, percent freezing was calculated as the number of times each animal was observed to be immobile over 8 observations. For context testing, percent freezing was calculated as the number of times each animal was observed to be immobile over 64 observations

### Wheel-running behavior analysis

The mice were housed individually and their wheel-running activity was recorded as revolutions per 3 min interval as previously described [32]. The running wheels and data acquisition system were obtained from Mini Mitter Co. (Bend, OR). The mice were first exposed to a 12:12 LD cycle for 2–3 weeks (light intensity 36 μW/cm^2 ^≅ 120 lux). The mice were then placed into constant darkness (DD) to assess their free-running activity pattern. Circadian time (CT) was determined by activity records with activity onset denoted as CT12. Training in DD was performed after 2–4 days in constant darkness. In the dark portion of LD conditions, as well as under DD conditions, handling of mice was carried out with the aid of an infrared viewer (FJW Industries, Ohio). Behavior during acquisition and recall was recorded with the aid of a video camera that had an in-built infrared system, which enabled us to record the animals in both light and dark conditions.

### Corticosterone measurements

Circulating corticosterone concentrations were determined 20 min after initiation of the weak training protocol. Trunk blood was collected by decapitation after isoflurane treatment. To obtain serum, blood samples were allowed to clot at room temperature for 30 min prior to centrifugation at 1000 × *g*. The supernatant was stored at -20°C until assayed. Samples were typically diluted 100-fold prior to assay. Serum corticosterone concentration was measured by competitive enzyme immunoassay (Correlate-EIA Corticosterone kit, Assay Designs, Michigan). The intra-assay CV was <8%, the inter-assay CV was <13.1% and the sensitivity was 27 pg/ml.

### *In Situ* Hybridization

A plasmid (pCRII, Invitrogen) containing the cDNA for mPer2 (9–489 nt, accession number AF035830) was generously provided by Dr. D. Weaver (Univ. Mass.) and insert identity was confirmed by sequencing using the M13R primer. To generate antisense and sense templates for ISH, plasmids were linearized overnight, phenol:chloroform extracted, ethanol precipitated and resuspended in DEPC-treated water. Riboprobes were synthesized from 1 μg of template cDNA in a reaction mixture containing 100 microCi of UTP ^35^S (1,250 Ci/mmol, Perkin Elmer, Wellesley, MA), 5× transcription buffer (Promega, Madison, WI), 0.1 M DTT (Promega, Madison, WI), 10 mM of each rATP, rCTP, rGTP, 40 U RNase Inhibitor, and the appropriate RNA transcriptase (SP6, or T7) for 3 hrs at 37°C. The *in vitro *transcription reaction was DNase I treated, then unincorporated nucleotides were removed using the RNase-free microfuge spin columns (Bio-Spin 30, Biorad, Hercules, CA) and probe yields were calculated by scintillation counting. ISH on tissue sections was done using previously described procedures (Lambert et al., 2005). Briefly, tissue sections were fixed in 4% paraformaldehyde, air-dried and blocked by acetylation with acetic anhydride, followed by a series of dehydration steps. After air drying, slides were placed in prehybridization buffer (50% formamide, 3 M NaCl, 20 mM EDTA, 400 mM Tris, pH 7.8, 0.4% SDS, 2× Denhardt's, 500 mg/mL tRNA, and 50 mg/mL polyA RNA) for 1 hr at 55°C. Sections were hybridized overnight at 55°C in humidified chambers in hybridization buffer (50% formamide, 10% dextran sulfate, 3 M NaCl, 20 mM EDTA, 400 mM Tris, pH 7.8, 0.4% SDS, 2× Denhardt's, 500 mg/mL tRNA, and 50 mg/mL polyA RNA, 40 mM DTT), where each slide was incubated with 1–4 million cpm/70 mL of a riboprobe. All post-hybridization washes contain 1 mM sodium thiosulfate, except in the RNase A and ethanol washes. Following hybridization, the slides were washed for 15 min in 4× SSC, at their respective hybridization temperatures, in 2× SSC for 1 hr at room temperature, then RNase A (20 micrograms/mL) treated at 37°C for 30 min to remove unbound probe. To further reduce non-specific hybridization, the slides were washed twice in 2× SSC at 37°C, and for 1 hr in 0.1× SSC at 62–67°C. Slides were serially dehydrated in ethanol containing 0.3 M ammonium acetate and exposed to Kodak Biomax MR film (Kodak, Rochester, NY) along with a ^14^C slide standard (American Radiolabeled Chemicals, St. Louis, MO). The slides were counterstained with 0.04% thionin dye to serve as a reference. Densitometric analysis of hybridization intensity was done as described using NIH image software [59].

### Statistical Analysis

To make simple comparisons between groups, t-tests were used. To compare recall for animals tested once at 24-, 48- or 72-hrs following training, one-way analysis of variance (ANOVA) was used followed by t-tests for pair-wise comparisons. In the cases in which repeated measurements were made from single animals, the data was analyzed using a one-way repeated measure (RM) ANOVA followed by Tukey's test for multiple comparisons. For all tests, values were considered significantly different at *P *< 0.05.

## Abbreviations

CS: conditioned stimulus; CT: circadian time; ISH: *in situ *hybridization; LD: light-dark; OD: optical density; SCN: suprachiasmatic nuclei; US: unconditioned stimulus; VIP: vasoactive intestinal peptide; WT: wild type; ZT: zeitgeber time.

## Authors' contributions

DC and AH carried out the fear conditioning experiments; DHL performed the corticosterone measurements and helped with the manuscript preparation; JMD carried out the ISH; CSC drafted the manuscript. All authors participated in the experimental design and approved the final manuscript.
